# Less activity means improved welfare? How pair housing influences pinyon jay (*Gymnorhinus cyanocephalus*) behaviour

**DOI:** 10.1017/awf.2024.40

**Published:** 2024-11-07

**Authors:** London M Wolff, Jeffrey R Stevens

**Affiliations:** B83 East Stadium, Department of Psychology, Center for Brain, Biology & Behavior, University of Nebraska-Lincoln, Lincoln, NE 68588, USA

**Keywords:** activity levels, animal welfare, bird, corvid, pair housing, stereotypies

## Abstract

The activity level and specific behaviours exhibited by captive animals are crucial indicators of welfare. Stereotypies, or repetitive behaviours that have no apparent function or goal, are performed by animals experiencing poor conditions in their environment and indicate welfare concerns. Changes in the housing environment in particular may have critical influences on behaviour and welfare. Here, we measured behavioural changes in a captive pinyon jay (*Gymnorhinus cyanocephalus*) population (n = 10) associated with a shift from single to pair housing. Using automated video processing, we show that pair housing greatly reduced overall activity levels in these birds. The stark reduction in activity was surprising, as we expected that social housing would increase interactions between birds, thus increasing activity levels. Upon further analysis, however, we found that stereotypic behaviours, such as beak scraping, jumping, pecking, and route tracing decreased after pair housing, whereas the positive welfare behaviours of perching and preening increased. Our results indicate that pair housing may reduce overall activity in pinyon jays; however, this reduction is primarily in stereotypic behaviours.

## Introduction

Bird owners use changes in behaviour to track well-being in birds, and a dramatic decrease in activity levels can indicate welfare problems. But could decreased behaviour actually be a sign of lower stress? Currently, activity/movement are offered as proxies for the welfare of an animal, with more activity typically linked to improved welfare (Tahamtani *et al.* 2019; Woods *et al.* 2022). However, when interpreting reduced activity levels, activity quality or type is rarely considered, highlighting a potential confound: if the behaviours that result in activity are themselves signals of stress, lower activity levels may paradoxically indicate better welfare. In this paper, we provide evidence of how pair housing of a social bird species is associated with decreased activity, but that the source of this change is decreased stereotypic behaviours, reflecting better, not worse, welfare.

Relative to other populations, captive animals are more likely to exhibit stereotypic behaviours, or repetitive behaviours that have no apparent function or goal (Mason 1991b). Stereotypies, sometimes referred to as abnormal repetitive behaviours, are performed by animals that have in the past or are currently experiencing poor conditions in their environment (Broom 1983; Mason 1991a,b; Mellor *et al.* 2018). Millions of captive birds — whether kept for companionship, education, production, or research — exhibit stereotypic or other abnormal repetitive behaviours (Mason & Latham 2004; Mason *et al.* 2007). These statistics are alarming as these behaviours are known to indicate welfare concerns (Mason 1991a,b; Rose *et al.* 2017). In birds, stereotypic behaviours include beak scraping, feather plucking, pecking, repetitive pacing, and route tracing (Garner *et al.* 2003; Mellor *et al.* 2018; Woods *et al.* 2022). Importantly, the presence of stereotypic behaviour can inform caretakers that welfare issues are a concern, but caution should be exercised when making causal assumptions, as there can be a lag between changes in the environment and the reduction of stereotypies if a reduction occurs at all.

Even though stereotypies are not causally interpretable, they typically indicate stress. It is therefore in the best interest of the animal for caretakers to apply stress-reducing strategies whenever stereotypies appear. Evidence-based solutions can help reduce or eliminate stereotypic behaviours, which is linked to welfare improvements (Mason & Latham 2004; Williams *et al.* 2018). The most widely used form of management to combat these abnormal behaviour patterns is environmental enrichment (Mason *et al.* 2007), with a meta-analysis showing that out of 41 studies, approximately half found significant reductions in stereotypy following implementation of environmental enrichment (Swaisgood & Shepherdson 2005). Other possible forms of intervention include punishment, genetic modification, and/or medication. However, these options do not address the underlying issues that cause stereotypies and, in some cases, may even increase or simply change the type of stereotypy an animal exhibits (Mason *et al.* 2007). Without addressing the underlying issue of housing or husbandry deficits that cause the stress, reducing stereotypic behaviours themselves is not an ideal endpoint.

One of the main underlying issues of housing or husbandry deficits in social animals is a lack of social interaction. Housing social species individually may induce stress resulting in stereotypies due to a lack of access to environments that allow the normal and natural functioning of their behaviours (Broom 1983; Swaisgood & Shepherdson 2005). This is especially true in social bird species where the connection between pairs of individuals is formed and strengthened through reciprocal preening and the exchange of food (Clayton & Emery 2007; Duque & Stevens 2016; Morales Picard *et al.* 2020; Miller *et al.* 2024).

For many social species, pair or group housing is recommended (Hawkins 2010; Baumans & Van Loo 2013). In a highly social bird species, parrots, social enrichment helps reduce stereotypies (Meehan *et al.* 2003; Williams *et al.* 2017). In fact, 57% of single-housed parrots (*Amazona amazonica*) developed stereotypies in the first 12 months of being housed in isolation, while pair-housed parrots developed no stereotypies (Meehan *et al.* 2003). Yet the manner of pairing is also critical. Forced pairings of male and female partridges (*Alectoris rufa*) resulted in more aggression and injuries than when females were allowed to choose their partner (Prieto *et al.* 2012). Thus, pair housing birds can confer benefits to welfare, but there are also welfare costs that require monitoring.

## Present study

Corvids comprise a family of birds found worldwide that includes ravens, crows, magpies, and jays. Due to their sophisticated cognition and varied social structures and feeding ecologies, corvids are a popular study species in the field of animal behaviour and cognition (Balda & Kamil 2002; Clayton & Emery 2007). With a number of research teams around the world maintaining corvids in captivity to study their behaviour, understanding their welfare is critical to this enterprise (Miller *et al.* 2024).

Here, we investigate the effects of housing practices on welfare for pinyon jays (*Gymnorhinus cyanocephalus*), a highly social corvid species that lives in mountainous regions of western North America (Marzluff & Balda 1992; Balda & Kamil 1998). Pinyon jays live in flocks ranging from 50 to 500 birds and experience frequent changes in the size and composition of their social groups (Marzluff & Balda 1992; Wiggins 2005). They exhibit sophisticated social behaviours, such as inferring transitive social relationships (Paz-y-Miño-C *et al.* 2004), exhibiting social learning (Templeton *et al.* 1999), food sharing (Duque & Stevens 2016), prosocial behaviour (Duque *et al.* 2018), and attending to competitor behaviour (Vernouillet *et al.* 2021).

Our colony of pinyon jays has historically been housed individually to maintain careful control of food intake (both in terms of food restriction and ensuring adequate access to food), which influences experimental motivation and performance as well as nutrition. However, given the need for social enrichment in corvids (Miller *et al.* 2024), we moved to pair house our birds. Pair housing balances the welfare needs of social housing with the logistic needs of ensuring food intake, easing the capture of birds for experimental sessions, and controlling exposure to conspecifics for social experiments. Pairs were fixed throughout this change to maintain consistent social relationships and to avoid confounding factors associated with multiple new partners. We therefore leveraged this change in housing to investigate the effects of different housing conditions on pinyon jay welfare as defined by activity and behaviour changes. We hypothesised that moving the birds to larger cages with a conspecific would result in more activity overall due to the new opportunity for social interactions.

Since manually observing and recording behaviours live is so time intensive (Rushen *et al.* 2012; Whitham & Miller 2016) and could disturb the birds if an observer is present, we video-recorded our pinyon jays before, during, and after they moved to new housing. We then employed automatic video analysis to quantify activity patterns by tracking pixel changes in the video images. After quantifying their overall activity, we then viewed the video and recorded specific behaviours that the birds exhibited, allowing us to map overall activity onto specific behaviours across the housing transition. We categorise those behaviours as positive or negative for welfare based on previous literature. Stereotypical/negative welfare behaviours in birds include beak scraping, pecking, repetitive jumping, and route tracing (Garner *et al.* 2003; Mellor *et al.* 2018; Woods *et al.* 2022); whereas foraging, perching, playing, and preening indicate positive welfare (Papageorgiou *et al.* 2023).

## Materials and methods

### Ethical approval

All procedures were conducted in an ethical and responsible manner and were approved by the University of Nebraska-Lincoln Institutional Animal Care and Use Committee (protocol number 2059), conforming to the ASAB/ABS Guidelines for the use of animals in research.

### Study animals

Our study population included 12 (three female) pinyon jays. On moving day, two male birds were replaced with two other males from a different housing room due to unrelated husbandry concerns. As a result, we only focused on the ten birds that completed all phases of the study when measuring individual behaviour (Table 1).

**Table 1.** Individual subject information

Subject	Sex	Capture year	Age at capture	Capture location*	Age at testing	Mean weight pre-move (g)	Mean weight post-move (g)
Piper	Female	2009	Adult	CA	14	95.7	95.9
Sapphire	Female	2009	Adult	CA	14	88.0	90.0
Uno	Female	2009	Juvenile	CA	14	89.5	90.7
Comanche	Male	2011	Juvenile	CA	12	107.5	108.2
Dartagnan	Male	2011	Unknown	CA	12	98.5	101.6
Prudence	Male	2011	Juvenile	CA	12	102.2	102.1
Dumbledore	Male	2010	Unknown	AZ	13	99.4	102.2
Fozzie	Male	2009	Adult	CA	14	103.4	103.9
He-man	Male	2009	Adult	CA	14	106.9	107.9
Fern	Male	2006	Unknown	AZ	17	94.9	99.1

* AZ = Arizona, CA = California

All birds were wild born, captured in either Arizona or California (United States Fish and Wildlife permit MB694205) between 2006 and 2011. At capture, they were estimated to be between one and three years of age. The birds in this study ranged in age from ten to 17 years with a mean of 13.1 years. During their time in the laboratory, all subjects experienced non-invasive cognitive and behavioural experiments and were handled by humans regularly. These experiments included studies of decision-making, numerical cognition, problem-solving, and social interactions (Paz-y-Miño-C *et al.* 2004; Bond *et al.* 2007; Duque & Stevens 2016; Stevens *et al.* 2016; Duque *et al.* 2018; Wolff *et al.* 2024).

## Procedures

### Housing

**Figure 1.**
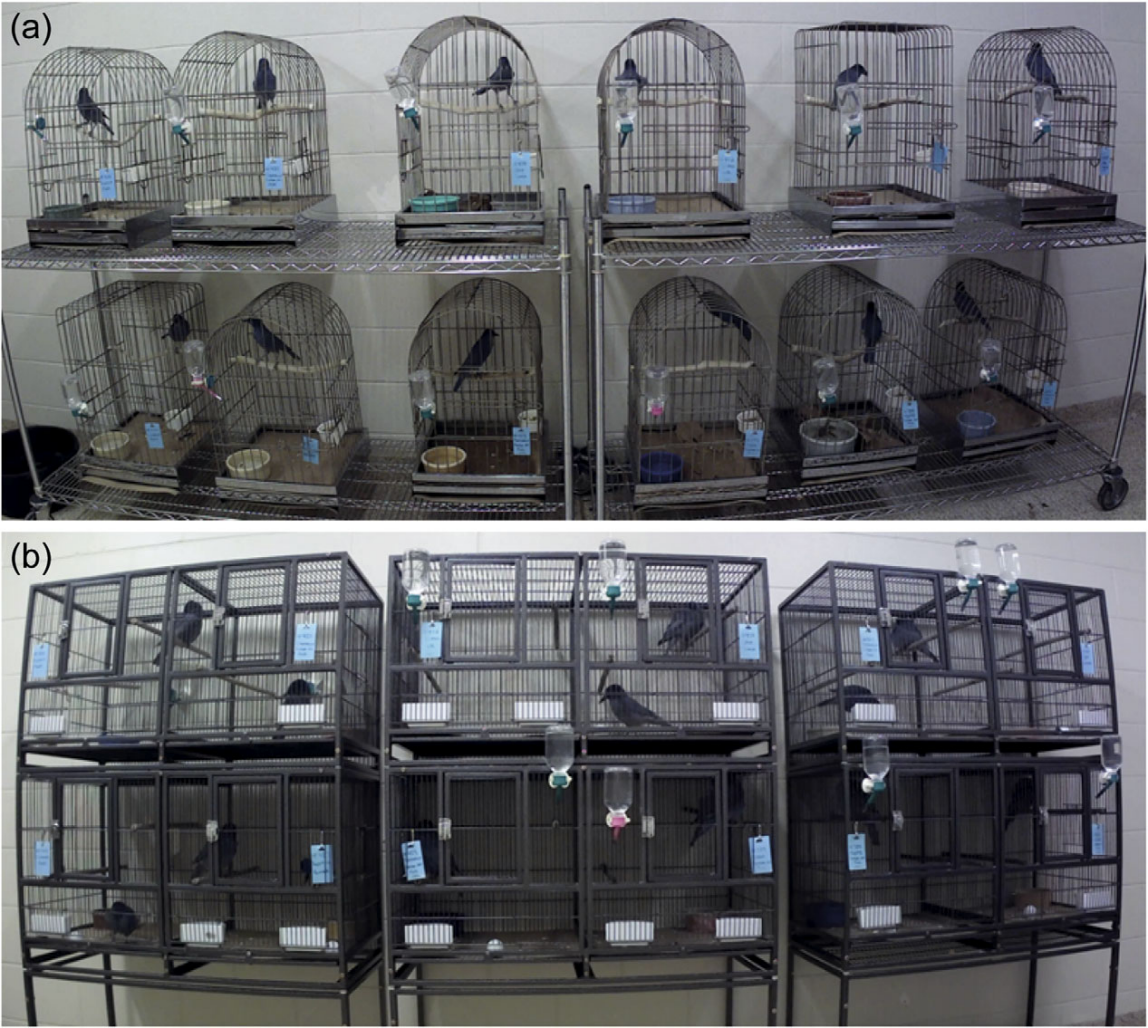
Camera screenshots of pinyon jays (*Gymnorhinus cyanocephalus*) in their cages showing (a) single and (b) pair housing. Figure used with permission under a CC-BY 4.0 licence: Wolff and Stevens (2024), available at https://doi.org/10.1101/2024.02.07.579343.

Data were collected over a three-week period between February 15th, 2021 and March 7th, 2021. During the first week, birds were kept in the single (0.10 m^3^) cages that they had been housed in upon entry to the colony (42 × 42 × 60 cm [length × width × height]; Figure 1[a]). After the first week, we moved each individual animal to their new larger cage (0.46 m^3^) with another bird (46 × 96 × 105 cm; Figure 1[b]). Birds were housed together based upon size, previous interactions, and sex. Where possible, we created female/male pairs as we have found that they are less likely to have behavioural issues together. The pairs were fixed throughout the study period. We label the first week as the *pre-move phase*, the second week as the *during-move phase*, and the third week as the *post-move phase.*

On moving day (February 22nd, 2021), the pinyon jays were placed on either side of the new cage with a divider in place to allow for the animals to acclimate to each other. After an hour of acclimation, the dividers were removed. Laboratory staff then watched the pairs continuously for the following 20 min and periodically for a further 2 h to ensure that no animals exhibited aggression or stress. As there was no evidence of negative interactions during this observation period, birds were allowed to remain with their original partners. Of the five pairs created, three were male/female and two were male/male. No enrichment was provided to the birds during the three phases of this study to prevent movement from objects being mistakenly recorded as bird movement. Following this study, we implemented enrichment by rotating through various toys and foraging tasks.

### Recording

We conducted 15-min video recordings of subjects in their home cage during the three-week study period 2–5 times per day (mean: 3.7× per day) between 0900 and 1700h CST. All recording occurred during the light phase within the rooms with a 14:10 h light:dark cycle. In the first week of recording, the animals resided in their original single housing, whereas in the subsequent two weeks, they were in the new pair housing. Three days prior to the first recording, we habituated the birds to the presence of a tripod and blue-coloured tape markings on the floor to signal the tripod’s location.

For recording sessions, an experimenter placed the camera (GoPro HERO9 Black; GoPro, San Mateo, CA, USA) on the tripod, turned on the camera, and left the room. No one entered the room during recording sessions. After 15 min had elapsed, the experimenter re-entered the room, turned off and removed the camera (leaving the tripod), and stored the video recordings. For pair housing, the tripod was adjusted to account for the new height of the paired cages; there were no other changes made to the recording protocol.

### Video processing and analysis

#### Activity levels

To quantify the amount of activity, we used a MATLAB script that calculated the sum of pixel changes across successive frames using the estimateFlow() function from the Computer Vision Toolbox®. The code started analysing frames 45 s into each video (to eliminate extraneous movement from the birds reacting to the experimenter turning on the camera) and ran until 10 min of video had elapsed. Three videos were removed from the analysis due to staff entering the housing room during recording. In total, 74 videos with 10 min of footage were used in the activity level analysis.

#### Behaviour data collection

To further investigate how specific behaviours changed over the three weeks, we coded the birds’ behaviours during week one and three. The first author (LW) created an ethogram of 18 behaviours that were present during the recordings: beak scraping, drinking, feeding, flapping, foraging, head through bars, hopping, jumping, laying down, other, out of view, pecking, perching, playing, preening, route tracing, standing, and walking (for behaviour definitions, see Table 2).

**Table 2.** Ethogram of pinyon jay (*Gymnorhinus cyanocephalus*) behaviours used to code video

Behaviour	Definition
Beak scraping	Bird runs its beak repeatedly back and forth along a branch.
Drinking	Bird’s head is raised toward water bottle or lowered to the water dish and has beak in contact with the water.
Feeding	Bird has its beak inside feeding cup.
Flapping	Bird is in an upright position and extends its wings repeatedly.
Foraging	Bird pecks or scratches at the ground or manipulates food items. Often includes moving paper in search of food items; however, bird is not only manipulating paper.
Head through bar	Bird has entire head through the bars of their cage stretching to the outside (can occur within a route-tracing bout).
Hopping	Bird jumps up and down on a solid object. Often occurs repeatedly, and it is not using hop to locomote.
Jumping	Bird jumps from one perch to another not in a route-tracing bout.
Laying down	Bird lays mostly calm and immobile on the floor.
Other	Any behaviour not belonging to the other categories.
Out of view	Bird is not visible for longer than 4s.
Pecking	Bird repeatedly pecks at their own body (leg band, back, feather, shoulder, etc.).
Perching	Bird’s feet grasp an elevated perch and bird is not locomoting.
Playing	Bird pecks or manipulates an object in the cage other than food or water dish. Unlike foraging it is not directed at searching for food. This can happen while the bird is moving or stationary.
Preening	Bird uses its beak to peck, stroke, or comb its own plumage.
Route tracing	Bird follows precise and consistent route within its cage (similar to pacing).
Standing	Bird maintains upright position on motionless, extended legs on the floor.
Walking	Low-speed movement of bird where there legs (not wings) are creating the movement.

Table used with permission under a CC-BY 4.0 licence: Wolff and Stevens (2024); available at https://doi.org/10.1101/2024.02.07.579343.

In the post-move phase two birds were present per cage (Figure 1). As individuals were difficult to identify, it was not possible to tell which of the paired birds were performing a specific behaviour. Therefore, we only coded whether a behaviour was present in either bird in a cage. To stay consistent across the phases, we also combined both of the birds that would eventually be housed together when coding pre-move phase video data. That is, we combine the behavioural data for each pair throughout both phases. Additionally, our analysis was limited to ten out of the 12 birds as only ten birds remained unchanged across phases.

For the behavioural analyses, we trimmed the videos to 10 min to match the activity level data. We then sampled a 10-s clip per minute per video. The first sample began at the 45-s mark and ended at the 55-s mark. The second sample began at 1 min 45 s and so on, until 9 min 45 s. We coded 20 recordings from the pre-move phase and 14 recordings from the post-move phase (we did not code any recordings from the during-move phase). We coded ten samples per pair per video, resulting in a total of 1,700 samples.

For each of the 18 behaviours on the ethogram, coders recorded the number of times that either bird in a pair exhibited each behaviour within every sample. Three coders coded the 1,700 samples. To ensure inter-rater reliability, prior to coding the full set, the three coders scored a test set of four videos. LW was aware of the response variable, but the other two coders were unaware. After training on the ethogram and common issues in coding, each coder received the same randomised subset of four videos to code. We calculated the intra-class correlation of their coded responses using a two-way random effects model for the average of three coders (ICC2k). Based on interpretations from Koo and Li (2016), the intra-class correlation demonstrated good reliability between raters (0.89). To score the full set of videos for analysis, the two unaware coders each scored half of the remaining videos.

## Data analysis

We used R (Version 4.4.0; R Core Team 2024) and the R-packages *BayesFactor* (Version 0.9.12.4.7; Morey & Rouder 2024), *cocoon* (Version 0.0.0.9000; Stevens 2024), *easystats* (Version 0.7.1; Lüdecke *et al.* 2022), *here* (Version 1.0.1; Müller 2020), *lme4* (Version 1.1.35.3; Bates *et al.* 2015), *papaja* (Version 0.1.2; Aust & Barth 2023), *patchwork* (Version 1.2.0; Pedersen 2024), *psych* (Version 2.4.3; Revelle 2024), and *tidyverse* (Version 2.0.0; Wickham *et al.* 2019) for our analyses. The manuscript was created using *knitr* (Version 1.46; Xie 2015), *kableExtra* (Version 1.3.4.9000; Zhu 2023), *rmarkdown* (Version 2.27; Xie *et al.* 2018), and *papaja* (Version 0.1.2; Aust & Barth 2023). Data, analysis scripts, supplementary materials, and reproducible research materials are available at the Open Science Framework (https://osf.io/v9r6q/).

Though we present both Bayesian and frequentist statistics (i.e. *P*-values), we draw inferences based on Bayes factors because they offer bidirectional information about evidence supporting both the alternative (H_1_) and the null (H_0_) hypotheses. Bayes factors provide the ratio of evidence for H_1_ over evidence for H_0_ (Wagenmakers 2007; Wagenmakers *et al.* 2010). Therefore, a Bayes factor of 3 (*BF*_10_ = 3) indicates three times more evidence for H_1_ than H_0_, whereas a Bayes factor of 1/3 (the reciprocal of 3) indicates three times more evidence for H_0_ than H_1_. We interpret Bayes factors based on Wagenmakers *et al.* (2018), where a *BF*_10_ > 3 is considered sufficient evidence for the alternative hypothesis, *BF*_10_ < 1/3 is considered sufficient evidence for the null hypothesis, and 1/3 < *BF*_10_ < 3 indicate neither hypothesis has evidence supporting it (suggesting the sample size is too small to draw conclusions).

### Activity levels

We estimated our response variable of activity level by calculating a mean number of pixel changes between video frames. To test the change in activity level over the different phases, we used model selection on linear models calculated with the lm() function. We then derived Bayes factors for comparing models from model BIC values using the test_performance() function from the *performance* package (Lüdecke *et al.* 2021). This approach implicitly assumes a unit information prior. Although we were primarily interested in the effect of phase on activity level, we also included time of day as a potential factor since activity may vary throughout the day. Therefore, we compared five models: (1) an intercept only model lm(activity ~ 1); (2) a phase only model lm(activity ~ phase); (3) a time of day only model lm(activity ~ timeofday); (4) a phase and time of day with no interaction model lm(activity ~ phase + timeofday); and (5) a phase and time of day with interaction model lm(activity ~ phase + timeofday) (Table 3). We calculated Bayes factors comparing each of the models with factors (models 2–4) to the intercept only model (1). We considered the model with the highest Bayes factor as the best fitting model.

**Table 3.** Model comparison for effect of phase and time of day on pinyon jay (Gymnorhinus cyanocephalus) activity level

Name	Model	AIC	BIC	BF
Intercept only	activity ~ 1	319.0	323.6	
Phase only	activity ~ phase	206.3	215.6	2.9×10^23^
Time of day only	activity ~ timeofday	320.6	327.5	0.14
Phase and time of day (main effects only)	activity ~ phase + timeofday	207.8	219.3	4.4×10^22^
Phase and time of day (interaction)	activity ~ phase * timeofday	210.7	226.8	1.0×10^21^

AIC = Akaike Information Criterion, BIC = Bayesian Information Criterion, BF = Bayes factor

### Behaviour data

For behaviour data, we calculated the mean frequency of each behaviour per pair for both the pre- and post-move phases. We then conducted frequentist and Bayesian paired *t*-tests to compare behaviour frequency across phases. For the Bayesian *t*-tests, we employed the ttestBF() function from the *BayesFactor* R package (Morey & Rouder 2024) using default priors (Cauchy distributions for effect sizes and noninformative/uniform distributions for variance).

## Results

### Activity levels

**Figure 2.**
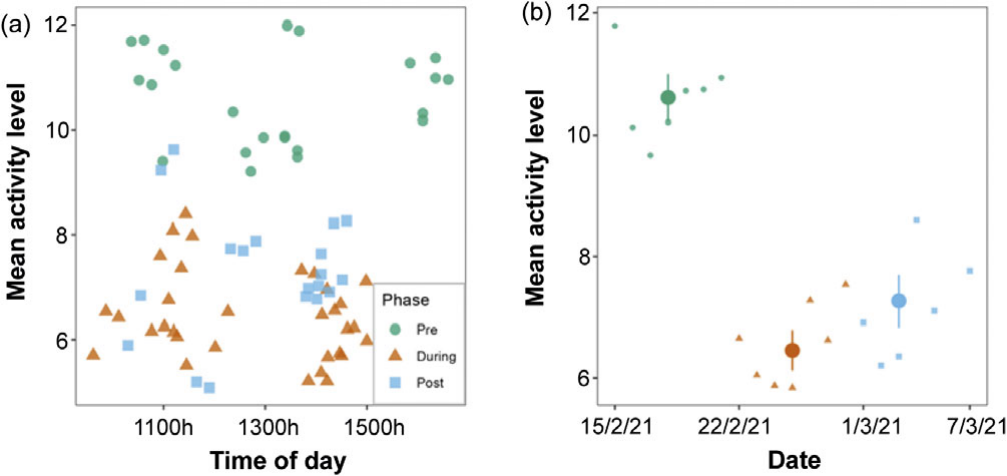
Activity levels of pinyon jays (*Gymnorhinus cyanocephalus*) before, during, and after moving from individual to pair housing (ten birds groups into five pairs). (a) Mean activity levels per sample across time of day for each phase. Points represent mean levels per individual video recording with phase indicated by colour and symbol. (b) Mean activity levels per sample across date. Points present mean levels averaged over dates with phase indicated by colour and symbol. Dots represent estimated marginal means per phase, and error bars represent 95% confidence intervals. Figure used with permission under a CC-BY 4.0 licence: Wolff and Stevens (2024); available at https://doi.org/10.1101/2024.02.07.579343.

Figure 2(a) shows the range of activity levels across time of day for the three phases. Our comparisons of models (Table 3) showed that the phase only model performed best. The phase only model had the highest Bayes factor (*BF*_10_ = 2.9 × 10^23^) compared to the time of day only model (*BF*_10_ = 0.14) and the phase and time of day model (*BF*_10_ = 4.4 × 10^22^). In fact, there was 6.5 times ore evidence favouring the phase only model over the next best (phase and time of day) model. Therefore, phase was an important predictor of activity levels, but time of day was not.

Since phase was important in predicting activity, we computed pair-wise contrasts for the different phases. These contrasts suggest that activity during the pre-move phase was substantially higher than both the during-move phase (mean difference = 4.15, *t*(71) = 16.1; *P* < 0.001, *d* = 3.8, *BF*_10_ = 3.0 × 10^20^) and post-move phase (mean difference = 3.34, *t*(71) = 11.4; *P* < 0.001, *d* = 2.7, *BF*_10_ = 1.2 × 10^10^). Further, activity levels increased slightly between the last two phases (mean difference = –0.81, *t*(71) = –2.9; *P* = 0.004, *d* = –0.7, *BF*_10_ = 7.3). Thus, changing housing greatly reduced overall activity levels (Figure 2[b]).

### Behaviour

**Figure 3.**
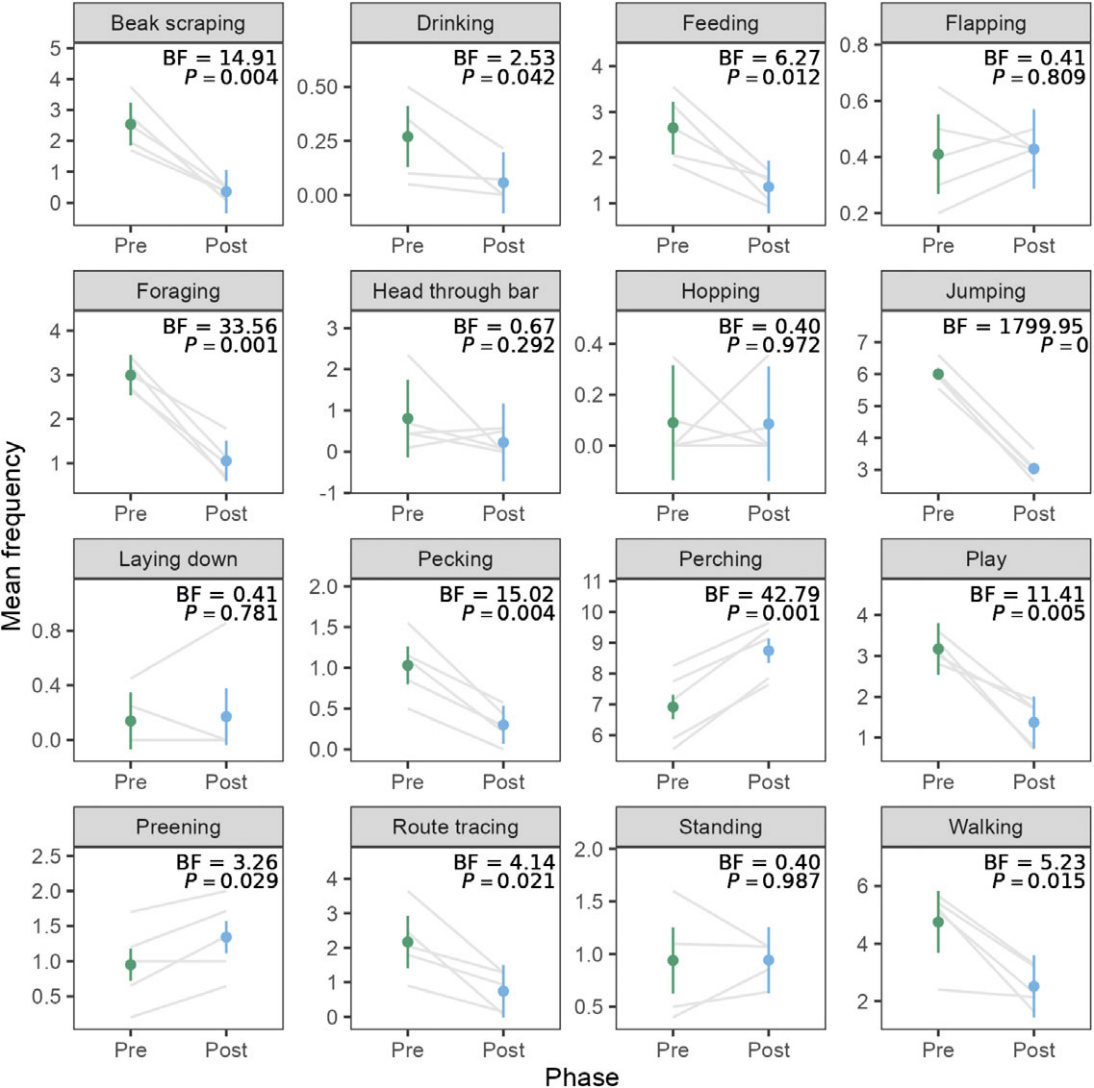
Mean frequencies of behaviours in the pre- and post-move phases for pinyon jays (*Gymnorhinus cyanocephalus*) (ten birds in five pairs). The behaviors other and out of view are omitted. Grey lines connect means for each of the five bird pairs. Dots represent overall means per phase, and error bars represent within-pair 95% confidence intervals. Figure used with permission under a CC-BY 4.0 licence: Wolff and Stevens (2024); available at https://doi.org/10.1101/2024.02.07.579343.

The stark reduction in activity was surprising, as we expected that social housing would increase interactions between birds, thus increasing activity levels. After uncovering this finding, we investigated the exploratory hypothesis that reduction in activity was driven by reductions in stereotypic behaviours. Figure 3 shows the mean frequencies for all of the behaviours, along with Bayes factors and *P*-values for the paired *t*-tests comparing frequencies in the pre- and post-move phases. Of the 18 behaviours, we observed a decrease in beak scraping, feeding, foraging, jumping, pecking, playing, route tracing, and walking. We observed an increase in perching and preening. We did not have enough evidence to detect differences or lack of differences in drinking, flapping, head through bar, hopping, laying down, or standing. We omitted analysis of behaviours other and out of view since they are not specific behaviours of interest.

### Weight

Throughout the study, we weighed our birds several times a week, so we could investigate the effects of the housing transition on this important measure of well-being (Labocha & Hayes 2012). Group housing in other wild-caught species has been shown to increase weight compared to individual housing (McLeod *et al.* 1997). Bird weights increased from the pre-move phase (*M* = 98.6 g, 95% CI [96.9, 100.3]) to the post-move phase (*M* = 100.2 g, 95% CI [98.8, 101.5]). A linear model with weights as the response variable and phase as a predictor outperformed an intercept-only model (*BF*_10_ = 148.4), indicating that phase influenced weight. Whether this weight increase was due to social facilitation or other aspects of pair housing is unclear. Regardless, moving to pair housing increased the birds’ weights, suggesting that their welfare improved.

## Discussion

We examined behavioural changes in pinyon jays during two husbandry interventions of a larger cage and pair housing. After the housing change, birds decreased their activity levels as measured by overall pixel changes during video recording. This dramatic drop in activity was surprising and motivated a more extensive follow-up analysis examining the frequencies of specific behaviours. This exploratory analysis indicated that perching and preening (considered positive welfare behaviours; Papageorgiou *et al.* 2023) increased in frequency after the cage change. In contrast, beak scraping, feeding, foraging, jumping, pecking, playing, route tracing, and walking decreased after the cage change. This reduction includes both stereotypic (beak scraping, jumping, pecking, route tracing) and positive behaviours (feeding, foraging, playing). Bird weights also increased following the housing change. Thus, moving to pair housing substantially altered behaviour in the pinyon jays with benefits to their welfare via reduced stereotypies, increased positive welfare behaviours, and increased weight.

The growing prevalence of automated behaviour assessment systems, such as video recording, accelerometers, and GPS devices can facilitate the large-scale collection of activity data (Rushen *et al.* 2012; Whitham & Miller 2016). However, researchers and animal caretakers must be mindful that overall patterns of activity do not necessarily provide a complete assessment of welfare. Measuring specific behaviours associated with stress and calm are critical to assessing welfare and formulating care plans. It is imperative to recognise that when employing activity measures as an indicator of welfare in captive animals, the absence of certain behaviours is not inherently problematic. Automated processes can be useful in assessing animal welfare, and improvements in technology such as computer vision may allow currently infeasible automation such as classifying and tracking individual behaviours. However, we argue that human observers provide an invaluable perspective on the welfare of captive animals.

### Study limitations

Despite our data providing intriguing insights into the effects of housing changes on captive bird welfare, we note several limitations of our study. First, this study involves a relatively small population of ten birds. Of course, individual differences are a critical component of animal behaviour and welfare (Stamps *et al.* 2012; Dingemanse & Wolf 2013; Richter & Hintze 2019). Interestingly, although some of the behaviours that we scored displayed a degree of variability, others remained relatively consistent. Beak scraping, foraging, jumping, and play all showed both consistent frequencies before the housing change and consistent drops in frequencies after the change. Other behaviours, such as foraging, pecking, perching, route tracing, and walking showed variability in the initial frequencies but consistent decreases (or increases) after the housing change. Thus, despite a relatively small sample size, most of our behavioural measures show consistent patterns across individuals. Moreover, the logistics of viewing videos of birds in pair housing did not enable us to identify and attribute behaviours to specific birds. Instead, behaviours were coded across bird pairs. Our findings are therefore limited to generalisations across pairs, rather than specific behavioural changes by a given individual. Larger samples with individually identifiable subjects would provide more confidence regarding the generalisability of results.

A second limitation is the advanced age of our birds (ten to 17 years old). There is very little work on behavioural changes that occur as birds age and none on age-related behavioural change in pinyon jays. What is considered an ‘older’ bird varies greatly within the existing literature (Collias *et al.* 1986; Anderson *et al.* 2004; Angelier *et al.* 2007; Class *et al.* 2019). One study found that older passerine birds rested significantly more than young adults (Collias *et al.* 1986). Thus, the older age of our birds might have resulted in more resting and less active behaviours, which could have reduced the effects of pair housing on more active behaviours. Also, younger birds show differing abilities in adapting to novel changes in their environments (Greenberg 2003). But this does not translate to older animals necessarily being more or less adaptive than younger ones (Dagg 2009). Therefore, more research is needed to understand the interaction between age, housing, and welfare.

It is possible that the reduction in activity and behaviours in our birds could have been an adverse reaction to the changes in housing. The lack of movement and increased perching could indicate more of a ‘freezing’ response to the stress of the change. While this is possible, the increase in preening indicates greater comfort with their surroundings (Papageorgiou *et al.* 2023). However, replicating this work with a larger sample size, a more diverse age range of birds, and perhaps more physiological measures of stress (e.g. cortisol, heart-rate levels) could clarify the effects of pair housing on bird welfare.

Finally, we only recorded behaviour for two weeks after the housing change. Alhough it was a small difference, activity levels in the third week increased compared to the second week. It is possible that the activity levels would have continued to increase over time. Therefore, we cannot claim that the behavioural differences observed here represent a sustained or permanent change in behaviour. Rather, we can only offer a snapshot in time that needs longer-term studies to determine if these activity patterns remain consistent as the pairs become more acquainted.

## Animal welfare implications

This study highlights a crucial distinction in the assessment of captive animal welfare: less activity does not necessarily imply poor welfare or increased stress. Rather, it is one facet of animal behaviour that must be examined when determining animal welfare. Our data in particular show that moving from single to pair housing can result in an overall reduction in activity. Yet that reduction does not occur uniformly across all behaviours. Our birds demonstrated reductions in stereotypic behaviours associated with stress, such as beak scraping, jumping, pecking, and route tracing. Therefore, the pair housing seems to have reduced these repetitive behaviours. However, it also decreased seemingly positive behaviours, such as foraging, playing, and walking. These behaviours might have decreased because the social enrichment associated with pair housing substituted for other forms of physical enrichment that the birds engaged in to maintain their own psychological welfare. Having a social partner present may have replaced the need to engage in these other activities. We also observed an increase in preening and perching. These behaviours may indicate a reduction in stress, where the animals feel comfortable enough in their environment that they can rest calmly and engage in self-care. However, there is less research on behaviours associated with improved welfare, so our subjective interpretations of these behaviours as positive may be biased. Overall, the move from single to pair housing seems to have reduced stress-related behaviours and potentially increased calming behaviours.

## Conclusion

This research investigated how pinyon jays showed paradoxically lower activity levels after moving from single to pair housing. Upon subsequent video analysis we found that the stereotypic behaviours of beak scraping, jumping, pecking, and route tracing decreased after pair housing, whereas a calming behaviour — preening — increased. Our findings suggest that pairing pinyon jays may decrease their overall activity, but this decrease is mainly observed in stereotypical behaviours. Further research is needed to determine whether this reduction in activity is sustained over time following initiation of pair housing.

## Data Availability

The data and analysis code are available at: https://osf.io/v9r6q/.
